# Supplementary material for: Effect of Animal Age, Postmortem Calcium Chloride Marination, and Storage Time on Meat Quality Characteristics of M. longissimus thoracis et lumborum of Buffalo Bulls
Source: Foods. 2022 Oct 13;11(20):3193. doi: 10.3390/foods11203193 (PMC9601457; doi:10.3390/foods11203193)
Supplement: Supplementary file 1 [file foods-11-03193-s001.zip › foods-1817065-supplementary.pdf]

Supplementary Table S1. Interaction effects of age groups, treatments and storage days on meat pH, color parameters (L*, a*, b*, C*, h* and ΔE) of *M. longissimus thoracis et lumborum* of buffalo bulls.

		pH	L*	a*	b*	C*	h*	ΔE
**Age groups x Treatments**								
**Young**	**Marinated**	5.80	47.01	16.31	6.38	17.59	21.27	2.34
	**Non- Marinated**	5.75	45.98	16.51	6.11	17.57	20.13	2.58
**Spent**	**Marinated**	5.60	46.54	15.82	8.03	17.93	26.84	2.73
	**Non- Marinated**	5.60	45.30	17.11	7.80	18.84	23.96	2.30
	**SE***	0.01	0.31	0.33	0.15	0.32	0.50	0.14
**Treatment x Days**								
**Marinated**	**Day 0**	5.64	47.74	18.37	7.91	20.41	22.44	0.77
	**Day 2**	5.60	47.55	15.45	7.11	17.25	24.95	2.99
	**Day 4**	5.69	46.86	16.01	7.05	17.57	24.02	2.44
	**Day 6**	5.72	46.94	15.77	7.17	17.46	24.66	2.65
	**Day 8**	5.75	45.74	16.09	7.34	17.74	23.65	2.71
	**Day 10**	5.81	45.83	14.71	6.63	16.14	24.62	3.99
**Non-Marinated**	**Day 0**	5.60	46.58	18.44	6.91	19.49	19.50	0.77
	**Day 2**	5.60	45.17	17.28	7.00	18.77	20.67	2.32
	**Day 4**	5.66	45.77	17.24	7.15	18.69	22.12	1.83
	**Day 6**	5.70	45.09	17.01	6.91	18.19	22.18	2.54
	**Day 8**	5.72	45.45	16.33	6.92	17.85	22.83	2.73
	**Day 10**	5.77	45.78	14.57	6.85	16.26	24.97	4.11
	**SE***	0.02	0.55	0.58	0.26	0.55	0.87	0.22
**Age groups x Treatment x Days**								
**Young Marinated**	**Day 0**	5.70	47.72	15.16	5.44	16.37	19.58	3.83
	**Day 2**	5.73	47.57	15.77	6.03	16.89	20.94	3.00
	**Day 4**	5.83	46.84	16.71	6.52	18.00	21.27	1.94
	**Day 6**	5.83	46.53	16.95	6.56	18.34	20.97	1.79
	**Day 8**	5.82	46.76	17.21	7.21	18.67	22.77	1.27
	**Day 10**	5.88	46.68	16.08	6.53	17.32	22.12	2.53
**Young Non-Marinated**	**Day 0**	5.70	46.43	15.00	4.72	15.63	17.02	4.39
	**Day 2**	5.68	46.27	15.42	5.13	16.30	18.18	3.86
	**Day 4**	5.78	46.52	16.94	6.41	18.14	20.64	1.88
	**Day 6**	5.78	45.44	17.41	6.43	18.26	20.48	2.21
	**Day 8**	5.75	45.33	17.98	7.20	19.37	21.80	1.89
	**Day 10**	5.85	45.90	16.37	6.82	17.76	22.69	2.46
**Spent Marinated**	**Day 0**	5.58	47.78	21.58	10.40	24.46	25.31	4.41
	**Day 2**	5.47	47.53	15.13	8.20	17.61	28.96	3.38
	**Day 4**	5.55	46.89	15.32	7.59	17.15	26.78	3.09
	**Day 6**	5.62	47.36	14.60	7.79	16.60	28.35	3.82
	**Day 8**	5.68	44.73	14.97	7.48	16.82	24.54	4.20
	**Day 10**	5.75	44.99	13.36	6.74	14.97	27.13	5.53
**Spent Non-Marinated**	**Day 0**	5.52	46.73	21.90	9.12	23.35	22.00	3.92
	**Day 2**	5.52	44.08	19.16	8.88	21.24	23.16	3.50
	**Day 4**	5.55	45.04	17.55	7.89	19.24	23.61	2.33
	**Day 6**	5.63	44.75	16.62	7.41	18.12	23.90	2.99
	**Day 8**	5.70	45.58	14.69	6.66	16.34	23.87	4.10
	**Day 10**	5.70	45.68	12.79	6.89	14.77	27.27	5.82
	**SE***	0.03	0.77	0.81	0.36	0.79	1.24	0.21

Means in the same column with different small letters (a,b,c,d,e,f) are significantly different ($p<0.05$). * SE: standard error.

Supplementary Table S2. Interaction effects of age groups, treatments and storage days on sensory attributes[#] (odor, flavor, texture and juiciness) of *M. longissimus thoracis et lumborum* of buffalo bulls.

		**Odor**	**Flavor**	**Texture**	**Juiciness**
**Age groups x Days**					
**Young**	**Day 2**	5.88	5.93	5.75	5.75
	**Day 10**	5.71	5.86	5.79	5.83
**Spent**	**Day 2**	6.31	6.21	5.66	5.56
	**Day 10**	6.26	5.90	5.78	5.70
	**SE**	0.10	0.09	0.08	0.09

Means in the same column with different small letters (a,b,c) are significantly different ($p<0.05$). * SE: standard error. # 1 = extremely non-beef-like odor, weak flavor, tough texture, dry in term of juiciness; 8 = extremely beef-like odor, strong flavor, tender texture, juicy.
